# Discovery of Depression-Associated Factors From a Nationwide Population-Based Survey: Epidemiological Study Using Machine Learning and Network Analysis

**DOI:** 10.2196/27344

**Published:** 2021-06-24

**Authors:** Sang Min Nam, Thomas A Peterson, Kyoung Yul Seo, Hyun Wook Han, Jee In Kang

**Affiliations:** 1 Department of Ophthalmology CHA Bundang Medical Center CHA University Seongnam Republic of Korea; 2 UCSF REACH Informatics Core, Department of Orthopaedic Surgery Bakar Computational Health Sciences Institute University of California San Francisco San Francisco, CA United States; 3 Department of Ophthalmology, Institute of Vision Research Eye and Ear Hospital, Severance Hospital Yonsei University College of Medicine Seoul Republic of Korea; 4 Department of Biomedical Informatics CHA University School of Medicine CHA University Seongnam Republic of Korea; 5 Department of Psychiatry Institute of Behavioral Science in Medicine Yonsei University College of Medicine Seoul Republic of Korea

**Keywords:** depression, epidemiology, machine learning, network, prediction model, XGBoost

## Abstract

**Background:**

In epidemiological studies, finding the best subset of factors is challenging when the number of explanatory variables is large.

**Objective:**

Our study had two aims. First, we aimed to identify essential depression-associated factors using the extreme gradient boosting (XGBoost) machine learning algorithm from big survey data (the Korea National Health and Nutrition Examination Survey, 2012-2016). Second, we aimed to achieve a comprehensive understanding of multifactorial features in depression using network analysis.

**Methods:**

An XGBoost model was trained and tested to classify “current depression” and “no lifetime depression” for a data set of 120 variables for 12,596 cases. The optimal XGBoost hyperparameters were set by an automated machine learning tool (TPOT), and a high-performance sparse model was obtained by feature selection using the feature importance value of XGBoost. We performed statistical tests on the model and nonmodel factors using survey-weighted multiple logistic regression and drew a correlation network among factors. We also adopted statistical tests for the confounder or interaction effect of selected risk factors when it was suspected on the network.

**Results:**

The XGBoost-derived depression model consisted of 18 factors with an area under the weighted receiver operating characteristic curve of 0.86. Two nonmodel factors could be found using the model factors, and the factors were classified into direct (*P*<.05) and indirect (*P*≥.05), according to the statistical significance of the association with depression. Perceived stress and asthma were the most remarkable risk factors, and urine specific gravity was a novel protective factor. The depression-factor network showed clusters of socioeconomic status and quality of life factors and suggested that educational level and sex might be predisposing factors. Indirect factors (eg, diabetes, hypercholesterolemia, and smoking) were involved in confounding or interaction effects of direct factors. Triglyceride level was a confounder of hypercholesterolemia and diabetes, smoking had a significant risk in females, and weight gain was associated with depression involving diabetes.

**Conclusions:**

XGBoost and network analysis were useful to discover depression-related factors and their relationships and can be applied to epidemiological studies using big survey data.

## Introduction

### Importance of Depression

Depression is a common debilitating psychiatric condition characterized by a low-spirited mood, loss of interest, and a range of emotional, cognitive, physical, and behavioral symptoms. It has a high global disease burden and had been projected to become the second most common cause of disability-adjusted life years worldwide by 2020 [1]. Thus, the determination of risk factors causing depression could have important implications in its prevention and intervention efforts by reducing modifiable risk factors. However, proper prevention and treatment of depression have been difficult owing to heterogeneity in the etiology and pathophysiology of depression [2].

### Conventional Modeling for Depression

Given the complex biological, psychological, and sociocultural factors underlying the pathogenesis of depression, an integrated model with confounder adjustment may provide a better understanding and multifaceted individualized approach for depression. In a typical study, survey-weighted logistic regression is used to identify depression-associated factors. A simple regression model for each candidate factor is built to adjust for age and sex. Then, a complex model is presented by adding more covariates to control potential confounders [3].

### Principal Factor Identification Problem in Conventional Modeling

A confounder has associations with both exposure and disease but is not in the causal pathway between exposure and outcome [4]. Confounding is a mixing of effects that obscures the real effect of exposure [4]. We might fit a regression model on all the measured potential confounders and minimize the confounding risk [5]. However, too many variables can cause multicollinearity or redundancy and make a regression model unstable with a high coefficient variance [5,6]. In contrast, variable selection may cause the exclusion of essential confounders [5]. Identification of the best subset of variables is challenging when the number of explanatory variables is large or when multicollinearity is present within the data [7].

### Benefits of Machine Learning–Based Modeling for Big Survey Data

For big survey data with numerous variables, the machine learning model can detect principle factors for a condition by attempting to maximize its predicting performance. During the training, dimensionality reduction (which reduces the number of features) is essential because many features may cause overfitting, and the generated model may not be generalized appropriately [8]. Moreover, feature selection can resolve multicollinearity by removing redundant features from a group of highly correlated features [8]. Regularization is a more advanced machine learning algorithm that reduces model complexity and improves generalizability [8]. A popular regularization method is LASSO (L1 regularization; least absolute shrinkage and selection operator) [8], also called a shrinkage method, which can be applied to collinear confounders and has shown advantages over conventional methods [5].

### Advantages of the Extreme Gradient Boosting Algorithm and Strategies of This Study

We speculate that extreme gradient boosting (XGBoost) can help select an optimal subset of essential variables from a large volume of survey data. XGBoost is an advanced implementation of a gradient-boosting decision-tree algorithm. XGBoost has been used in a few studies to predict or screen depression [9-11]. XGBoost is advantageous because of its high speed and performance, making it dominant in applied machine learning for structured data. XGBoost also offers regularized gradient boosting and feature importance scores using a trained predictive model, which can be used for feature selection [12].

However, XGBoost has many hyperparameters that should be manually specified, and optimal parameter tuning can be challenging. To solve this problem, we have chosen an automated machine learning tool, the tree-based pipeline optimization tool (TPOT), along with genetic programming [13]. To the best of our knowledge, this study is the first attempt at an XGBoost-supported epidemiologic investigation regarding depression in the general population using national survey data.

## Methods

### Overview of Survey Data

**Figure 1:**
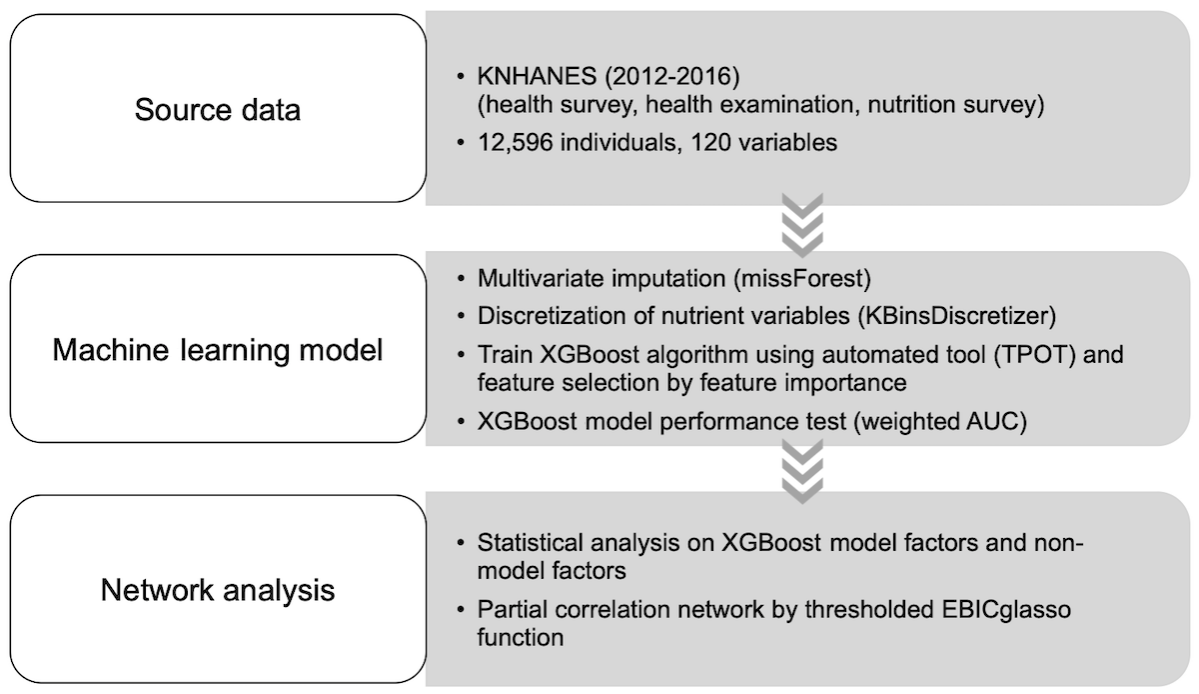
Flow diagram of the study. AUC: area under the curve; EBICglasso: extended Bayesian information criterium graphical lasso; KNHANES: Korea National Health and Nutrition Examination Survey.

The overall study flowchart is provided in Figure 1. We used survey data obtained from 12,596 Koreans (aged 19-64 years) who participated in the Korea National Health and Nutrition Examination Survey (KNHANES, 2012-2016), which is an annual survey conducted by the Korea Centers for Disease Control and Prevention (KCDC) in the Republic of Korea, wherein population-wide health and nutritional statuses are assessed [14]. The maximum age was set to 64 years because the food frequency survey was conducted for those aged 19 to 64 years. The design and methods of the KNHANES and the data resource profile are available in previous reports [14-16]. KNHANES V-3 (2012), KNHANES VI (2013-2015), and KNHANES VII-1 (2016) were approved by the KCDC research ethics committee (2012-01EXP-01-2C, 2013-07CON-03-4C, 2013-12EXP-03-5C, and 2015-01-02-6C), and written informed consent was obtained from all the subjects.

In the annual KNHANES (2012-2016) following a multistage clustered probability design, 192 primary sampling units (PSUs) were generated from approximately 200,000 geographically defined PSUs for the entire country; subsequently, 20 final target households were sampled for each PSU as secondary sampling units (ID_fam) [16]. The sample weights were calculated using the inverse of selection probabilities and the inverse of response rates that were adjusted to gender- and age-specific Korean populations (poststratification). Therefore, the sampled individuals accurately represented the Korean population.

### Depression Case Definition

Positive depression diagnosed by a physician (DF2_dg) and positive present depression (DF2_pr) represented the positive case “current depression,” and these indicated that the individual had a diagnosis of depression and was currently experiencing depression, respectively. In terms of the negative case, both DF2_dg and DF2_pr were negative and represented “no lifetime depression,” which indicated that the individual had never experienced depression. DF2_dg and DF2_pr were reported by the participants in this survey.

### XGBoost-Derived Model for Depression

We included as many variables as possible (Textbox 1) and imputed missing values using univariate imputation (filling with the last valid value for categorical or discrete variables) or multivariate imputation for missing continuous variables. The nutrient variables were discretized into three bins as follows: low, medium, and high. We converted categorical variables to numerical variables using one-hot encoding and dropped reference categories. We standardized variables, except for a binary dummy variable, by removing the mean and scaling them to unit variance before machine learning.

**Study variables of the Korea National Health and Nutrition Examination Survey data.**

**Health Examination Data**

Physical examination

- Waist circumference, BMI, regularity of pulse, and periodontal disease

Blood tests

- Anemia (hemoglobin <13.0 g/dL for men; hemoglobin <12.0 g/dL for nonpregnant women; hemoglobin <11.0 g/dL for pregnant women), white blood cell count, platelet count, red blood cell count, aspartate aminotransferase, alanine aminotransferase, creatinine, urea nitrogen, hepatitis B surface antigen, and hepatitis C antibody

Fasting (≥8 hours) blood tests

- Sugar level, high-density lipoprotein cholesterol (HDL), and triglyceride (TG)

Urine strip test

- pH, nitrite, specific gravity, protein, glucose, ketones, bilirubin, blood, urobilinogen

Pulmonary function test

Hypercholesterolemia: total cholesterol (TC) ≥240 mg/dL or lipid-lowering medication

Hypertension: systolic pressure ≥140 mmHg or diastolic pressure ≥90 mmHg or medication

Diabetes mellitus: fasting blood sugar level ≥126 mg/dL, diagnosis, medications, or insulin injections

Prediabetes: fasting blood sugar level ≥100 and <126 mg/dL

Low-density lipoprotein cholesterol (LDL) = TC – (HDL + TG/5), where TG ≤400 mg/dL

**Health Survey Data**

- Age, marital status, educational level, occupational class, household income, weight changes in the past year, alcohol consumption frequency, sleep hours per day, perceived stress, current smoking status, walking for more than 10 minutes (days/week), muscle-strengthening activities (days/week), childbirth experience, and EuroQol five-dimension three-level (EQ-5D-3L) questionnaire (mobility, usual activities, self-care, and pain/discomfort; three levels for each) [17]

Morbidity

- Chronic obstructive pulmonary disease, stroke, ischemic heart disease, osteoarthritis, rheumatoid arthritis, pulmonary tuberculosis, asthma, thyroid disease, gastric cancer, hepatoma, colon cancer, breast cancer, cervix cancer, lung cancer, thyroid cancer, atopic dermatitis, renal failure, hepatitis B, hepatitis C, and liver cirrhosis

Oral health

- Difficulty chewing, caries treatment within the last 1 year, root canal treatment within the last 1 year, and prosthetic treatment within the recent 1 year

**Food Frequency Survey Data (daily intake)**

- Carbohydrates, proteins, saturated fatty acids, monounsaturated fatty acids, polyunsaturated fatty acids, n3 fatty acids, n6 fatty acids, n6:n3 fatty acid ratio, cholesterol, fiber, vitamin A, vitamin B1, vitamin B2, vitamin C, niacin, iron, calcium, potassium, phosphorus, and sodium

The training set included 80% “current depression” and 80% “no lifetime depression” (10,076 cases). The remaining cases were assigned to the test set (2520 cases). We used TPOT to choose the best XGBoost hyperparameters from the training set to predict “current depression” from “no lifetime depression.” Then, the model features were selected further using the feature importance of XGBoost. The XGBoost model was explained using shapley additive explanation (SHAP) values that showed each feature’s impact on the model prediction [18].

The weighted area under the receiver operating characteristic curve (AUC) of the final model was calculated using the sample weight [19]. The optimal threshold was computed as the prediction probability at which Youden’s index (sensitivity + specificity – 1) was the maximum on the test set. We also estimated the weighted AUC of the model for the Patient Health Questionnaire-9 (PHQ-9) score (depression when score ≥10) instead of the reported depression.

### Statistical Test for Model Factors and Exploring Nonmodel Factors

Survey-weighted multiple logistic regression was performed using the model features from all samples (training and test sets). We defined the “direct” factor of depression as the model feature whose multiple logistic regression coefficient significantly differs from zero (*P*<.05); the other model features were designated as “indirect” factors. Subsequently, we performed a statistical test on factors excluded by the model to identify significant “nonmodel” factors that were not chosen by XGBoost as optimal for predicting depression. Nonmodel factors whose coefficients of multiple logistic regression with the model features were significantly different from zero (*P*<.05 with Bonferroni correction) were chosen.

### Network Analysis for Depression-Related Factors

A correlation matrix of model features and nonmodel factors was generated using all samples. Based on the correlation matrix, a thresholded EBICglasso (extended Bayesian information criterium graphical lasso) network was plotted, in which the network model was estimated using graphical LASSO regularization with EBIC model selection. Each edge weight was the correlation coefficient between two nodes after controlling all other network correlations [20,21].

Three centrality indices (strength, closeness, and betweenness) were computed. Strength centrality is the absolute sum of the edge weights connected to the node. Closeness centrality is the sum of the shortest distances from the node to all other network nodes. Betweenness centrality is the number of times when the node lies on the shortest path between two other nodes [21,22].

### Confounding or Interaction Effects on Indirect Risk Factors

If an indirect risk factor was positively connected to direct risk factors, the direct factor confounding effects were tested. If the indirect factor’s coefficient on survey-weighted multiple logistic regression became significant without the direct factors, we assumed they were the indirect factor’s confounders.

If an indirect risk factor was positively connected to a direct preventive factor or negatively related to a direct risk factor, the interaction effect was tested using the interaction term on survey-weighted multiple logistic regression.

### Statistics and Software

We used the following two programming languages: Python (version 3.8.5, Python Software Foundation) and R (version 3.6.3, R Foundation for Statistical Computing).

#### Scikit-Learn Tools

The following scikit-learn tools [23] were applied: for imputing missing continuous values, *IterativeImputer* class with the ExtraTreesRegressor estimator; for discretization, *KBinsDiscretizer* with a k-means strategy based on a k-means clustering procedure; for standardization, *StandardScaler*; and for weighted AUC, sensitivity, specificity, and threshold, *roc_auc_score* and *roc_curve*.

#### TPOT

TPOT starts from a collection of random models (first generation). Subsequently, those with higher performance are chosen and copied into the next generation’s population. The offspring crossover with other offspring or are randomly changed by mutation. The algorithm repeats this evaluate-select-crossover-mutate process for multiple generations. Finally, the best model is selected from the run [13].

We used TPOT to tune the hyperparameters of the XGBClassifier, such as the number of boosting rounds (“n_estimators”), boosting learning rate (“learning_rate”), maximum tree depth for base learners (“max_depth”), minimum sum of instance weight needed in a child (“min_child_weight”), minimum loss reduction required to make a further partition on a leaf node of the tree (“gamma”), subsample ratio of the training instance (“subsample”), subsample ratio of columns when constructing each tree (“colsample_bytree”), L1 regularization term on weights (“reg_alpha”), and L2 regularization term on weights (“reg_lambda”). To control the balance of positive and negative weights, we set “scale_pos_weight” as the number of negative cases/the number of positive cases. The “objective” option was set to a default value, “binary:logistic” (ie, logistic regression for binary classification).

Two parameters of TPOTClassifier, namely, number of iterations to run the XGBoost optimization process (“generation”) and number of individuals to retain in the genetic programming population every generation (“puplation_size”), were set to 100 (default) each. Finally, TPOT evaluated models using AUC (“roc_auc”).

#### Extreme Gradient Boosting (XGBoost) and Feature Selection

XGBoost incorporates an ensemble technique (boosting) of adding new models to correct errors made by previous models. When adding new models, gradient boosting uses a gradient descent algorithm to minimize the loss. More details on XGBoost’s working principles can be found in published articles [24]. For feature selection, we sorted the model features according to their importance (importance_type = “gain,” the average gain of splits that use the feature) and selected a subset of features by omitting the least important feature. If the AUC of the model with the subset of features was not compromised or became higher, we omitted the next least important feature. Subsequently, we retested it until the model performance reached the best AUC.

#### R Packages

The *Survey* package allows to specify a multistage sampling survey design and provides functions to estimate total population counts, means, and variances for a survey sample [25]. In this study, the survey design of the KNHANES was applied using the variables *psu* for PSU, *ID_fam* (family ID) for the secondary sampling unit, *kstrata* for strata, and sample weight (*wt_tot*/5) for weight. Additionally, we used the “svyglm” function from the *survey* package for survey-weighted multiple logistic regression. The odds ratio was calculated by exponentiating the coefficient of multiple logistic regression.

Receiver operating characteristic (ROC) curves were generated using the *ggplot2* package [26], and the weighted AUC was calculated by using the “WeightedAUC” function with the sample weight (*WeightedROC* package) [19].

#### Network Graph

For a correlation matrix in network analysis, a correlation between two variables was calculated using the wtd.cor function with the sample weight for “weight” in R’s weights package, which produced weighted Pearson correlation coefficients for survey data [27]. The network graph was generated using the qgraph function in the *qgraph* package of R by setting the graph argument to “glasso” and layout to “spring” with threshold [20]. For node sizes in the network, the effective size of the variable’s odds ratio was calculated by exponentiating the absolute coefficient of multiple logistic regression. For the calculation of centrality indices, the qgraph object and the centralityTable function in the *qgraph* package of R were used [20].

## Results

### XGBoost-Derived Model and the Performance

The population was estimated to be 22,262,880 (SE 355,977) (97.3%) for no depression reported during lifetime and 616,082 (SE 40,273) (2.7%) for current depression (complex-survey-design-based estimation). After TPOT training, the number of XGBoost model features was reduced from 120 to 81 (33% reduction) by the L1 regularization term of XGBoost. Furthermore, we decreased the feature number to 18 (78% reduction) by applying feature selection without performance loss.

**Figure 2:**
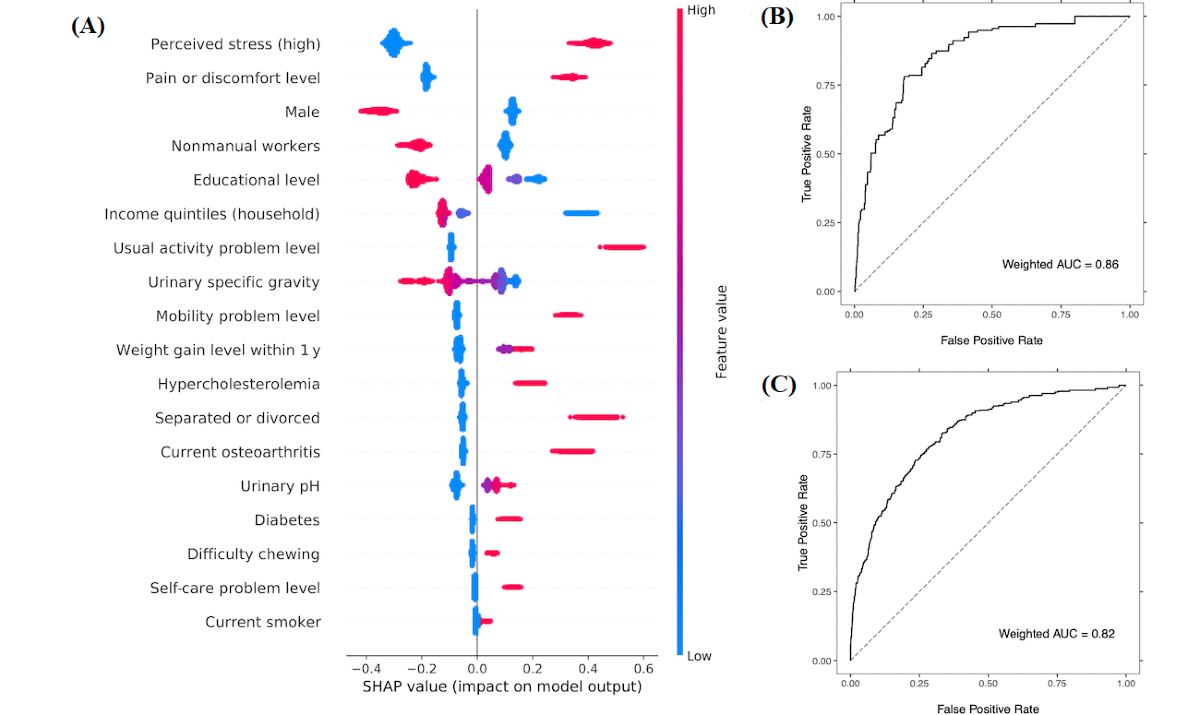
XGBoost model for the depression and performance test. Red dots represent positive for a binary factor or high feature values for a continuous factor on the beeswarm plot of shapley additive explanation (SHAP) values (log odds of the current depression) for the training data (A). On the weighted receiver operating characteristic curve (ROC) (B), sensitivity is 0.78 and specificity is 0.82 at the best threshold of probability of 0.461 for test samples. The model is also tested using the Patient Health Questionnaire-9 (depression when score ≥10) for 6098 samples (C). AUC: area under the curve.

The final model features included mental health, quality of life (QoL), socioeconomic factors, morbidity, sex, marital status, urinalysis, and health behavior (Figure 2). Nutrition factors failed to be included in the model. For the test set, the weighted AUC of the model was 0.86 for reported depression and 0.82 for PHQ-9 score depression (Figure 2). The model’s accuracy was 0.82 at the best threshold of the test data (Table 1).

**Table 1:** Confusion matrix for the test data set.

Actual depression	Predicted depression^a^	
	Current	No lifetime
Current	103,182	28,933
No lifetime	804,195	3,657,604

^a^Case numbers are estimated using complex-survey-design weights at the best threshold.

### Statistical Significance of Depression-Associated Factors

Perceived stress and current asthma were top-ranked statistically significant risk factors (Table 2). Triglycerides, current asthma, and farm workers were nonmodel factors having a significant association with depression and added on the network with model factors. Eight features out of 18 model factors were not statistically significant and classified as indirect (Table 2).

**Table 2:** Survey-weighted multiple logistic regression analysis of depression-related factors.

Variable			No lifetime depression^a^ (N=22,262,880)		Current depression^a^ (N=616,082)		Odds ratio (95% CI)^b^		*P* value
**High perceived stress (≥ 3/4 points), n (%)**									
	Yes	5,999,970 (26.95%)		372,138 (60.40%)		3.3 (2.5-4.3)		<.001	
	No (reference)	16,262,910 (73.05%)		243,944 (39.60%)		N/A^c^		N/A	
**Sex/gender, n (%)**									
	Male	11,072,884 (49.74%)		165,770 (26.91%)		0.5 (0.3-0.7)		<.001	
	Female (reference)	11,189,996 (50.26%)		450,312 (73.09%)		N/A		N/A	
**Marital status, n (%)**									
	Separated or divorced	852,170 (3.83%)		105,567 (17.14%)		2.2 (1.4-3.5)		<.001	
	Single	6,153,788 (27.64%)		133,952 (21.74%)		1.4 (0.9-2.2)		.11	
	Widowed	397,387 (1.78%)		36,923 (5.99%)		1.4 (0.8-2.6)		.25	
	Married (reference)	14,859,535 (66.75%)		339,640 (55.13%)		N/A		N/A	
**Occupation, n (%)**									
	Nonmanual	10,304,597 (46.29%)		161,758 (26.26%)		0.6 (0.4-0.8)		<.001	
	Manual	4,394,739 (19.74%)		102,028 (16.56%)		0.7 (0.4-1.0)		.06	
	Farm^d^	498,239 (2.24%)		10,848 (1.76%)		0.4 (0.2-0.8)		.02	
	Unemployed (reference)	7,065,305 (31.73%)		341,448 (55.42%)		N/A		N/A	
**Asthma^d^, n (%)**									
	Current	205,771 (0.92%)		34,769 (5.64%)		3.1 (1.5-6.5)		.002	
	Past	303,213 (1.36%)		10,387 (1.69%)		1.3 (0.5-3.2)		.57	
	No lifetime (reference)	21,753,896 (97.72%)		570,926 (92.67%)		N/A		N/A	
**Hypercholesterolemia, n (%)**									
	Yes	2,963,312 (13.31%)		146,742 (23.82%)		1.4 (0.9-1.9)		.10	
	No (reference)	19,299,568 (86.69%)		469,340 (76.18%)		N/A		N/A	
**Osteoarthritis, n (%)**									
	Current	825,130 (3.71%)		100,307 (16.28%)		1.4 (0.9-2.1)		.17	
	Past	155,266 (0.70%)		8,385 (1.36%)		0.7 (0.1-4.6)		.71	
	No lifetime (reference)	21,282,484 (95.59%)		507,390 (82.36%)		N/A		N/A	
**Diabetes, n (%)**									
	Yes	1,466,133 (6.59%)		84,663 (13.74%)		1.4 (0.9-2.2)		.11	
	Prediabetes	4,504,230 (20.23%)		125,783 (20.42%)		1.0 (0.7-1.4)		.81	
	No (reference)	16,292,517 (73.18%)		405,636 (65.84%)		N/A		N/A	
**Difficulty chewing, n (%)**									
	Yes	3,398,898 (15.27%)		173,323 (28.13%)		0.9 (0.7-1.3)		.65	
	No (reference)	18,863,982 (84.73%)		442,759 (71.87%)		N/A		N/A	
**Current smoker, n (%)**									
	Yes	5,178,518 (23.26%)		139,892 (22.71%)		1.2 (0.8-1.8)		.47	
	No	17,084,362 (76.74%)		476,190 (77.29%)		N/A		N/A	
Pain or discomfort level (1 to 3 points), mean (SE)			1.2 (0.004)		1.6 (0.034)		1.3 (1.1-1.5)		<.001
Fasting serum triglyceride (mg/dL)^d^, mean (SE)			134.8 (1.3)		161.8 (12.0)		1.2 (1.1-1.3)		.002
Weight gain level within 1 year (0 to 3 points), mean (SE)			0.4 (0.009)		0.6 (0.047)		1.2 (1.1-1.3)		.003
Urine specific gravity, mean (SE)			1.020 (0.00007)		1.018 (0.0004)		0.8 (0.7-0.9)		.003
Usual activity problem level (1 to 3 points), mean (SE)			1.0 (0.002)		1.3 (0.029)		1.1 (1.0-1.3)		.006
Income quintiles (household) (1 to 5 points), mean (SE)			3.4 (0.02)		2.6 (0.09)		0.8 (0.7-0.9)		.007
Educational level (1 to 4 points), mean (SE)			3.2 (0.01)		2.6 (0.06)		0.8 (0.7-1.0)		.01
Urine pH, mean (SE)			5.7 (0.009)		5.8 (0.050)		1.1(1.0-1.3)		.07
Mobility problem level (1 to 3 points), mean (SE)			1.1 (0.002)		1.3 (0.032)		1.1 (1.0-1.2)		.22
Self-care problem level (1 to 3 points), mean (SE)			1.0 (0.001)		1.1 (0.017)		1.0 (0.9-1.1)		.98
Age (years)^e^, mean (SE)			40.7 (0.2)		45.1 (0.8)		1.1 (0.8-1.3)		.61

^a^Population counts (n), means, and standard errors are estimated using complex-survey-design weights.

^b^For continuous factors, the value was standardized by removing the mean and scaling to unit variance.

^c^N/A: not applicable.

^d^Nonmodel factors were found by controlling for model features and age.

^e^Not a depression-related factor, but included to control confounding effects of age.

### High-Centrality Nodes and Clusters in the Depression Network

**Figure 3:**
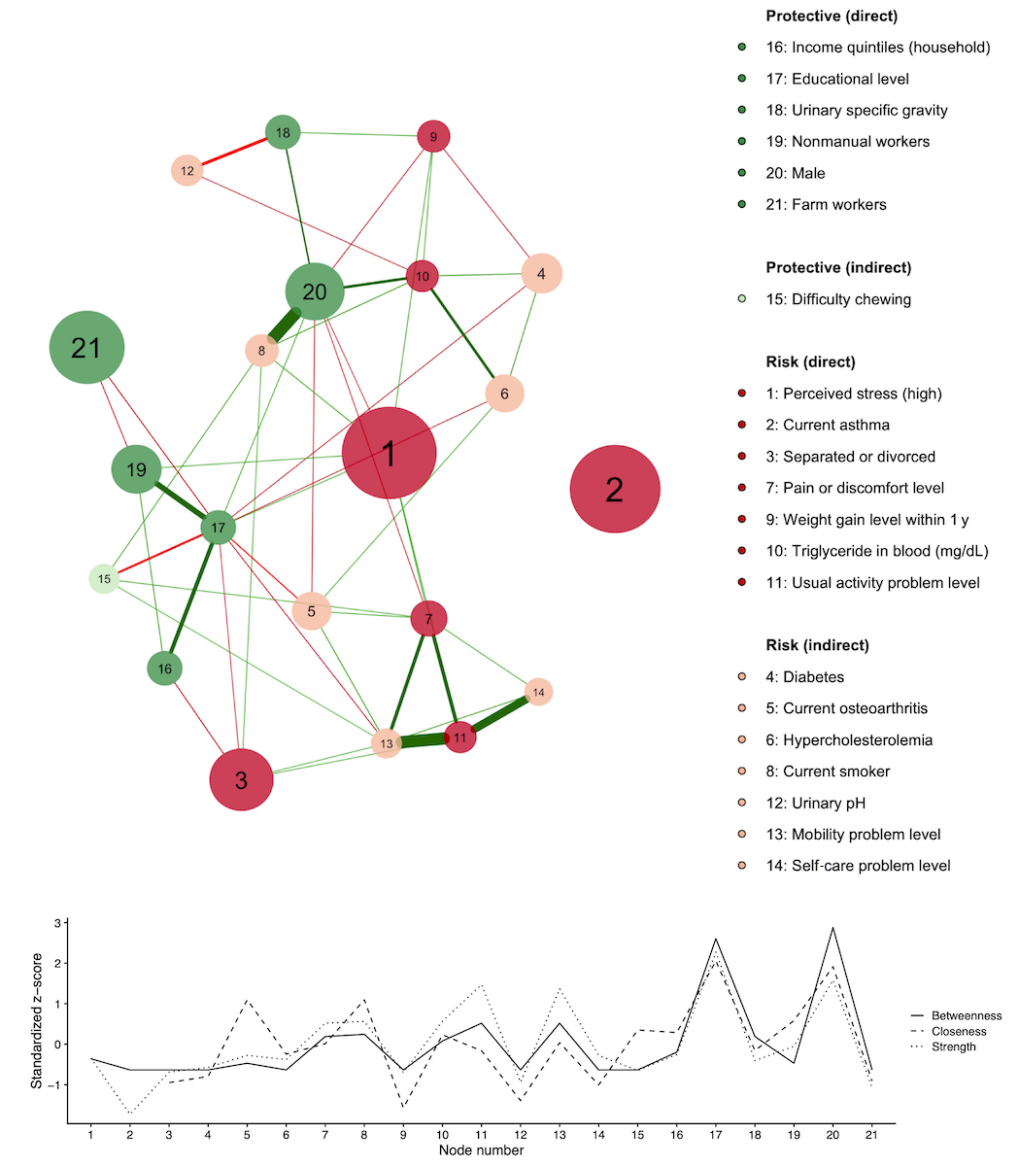
Partial correlation network graph and centrality indices for depression-associated factors. The factors can be positively (risk) or negatively (protective) related to current depression. Node size is proportional to the effective size of the odds ratio. Green and red edges represent positive and negative correlations, respectively. The edge with the highest absolute weight has full-color saturation and the widest width.

Based on the centrality indexes, the network had two high-centrality nodes, namely, “educational level” and “male,” which meant both nodes were highly connected with various factors (Figure 3). Some factors were strongly connected to form a cluster (eg, the socioeconomic status [SES] cluster consisted of nonmanual workers, farm workers, educational level, and household income) (Figure 3). The QoL cluster comprised four EuroQol five-dimension three-level (EQ-5D-3L) domains (“pain or discomfort level,” “usual activity problem level,” “mobility problem level,” and “self-care problem level”) (Figure 3).

### Confounders and Interaction Effects of Indirect Factors

Two indirect factors, namely, hypercholesterolemia and diabetes, were positively connected with triglyceride on the network, and triglyceride was a confounder of hypercholesterolemia and diabetes (Table 3). Another indirect factor, current osteoarthritis, became statistically significant after excluding its positive associates, namely, “pain or discomfort level” and “mobility problem level” (Table 3).

**Table 3:** Possible confounders on indirect factors in depression.

Indirect factors	Confounders^a^	Odds ratio (95% CI)		
		With confounders		Without confounders
Current osteoarthritis	Pain or discomfort level and mobility problem level		1.4 (0.9-2.1)	1.6 (1.1-2.5)
Hypercholesterolemia	Triglyceride		1.4 (1.0-1.9)	1.5 (1.0-2.1)
Diabetes	Triglyceride and hypercholesterolemia		1.4 (0.9-2.2)	1.7 (1.1-2.6)

^a^A group of confounders to make a coefficient of the indirect factor statistically insignificant.

**Figure 4:**
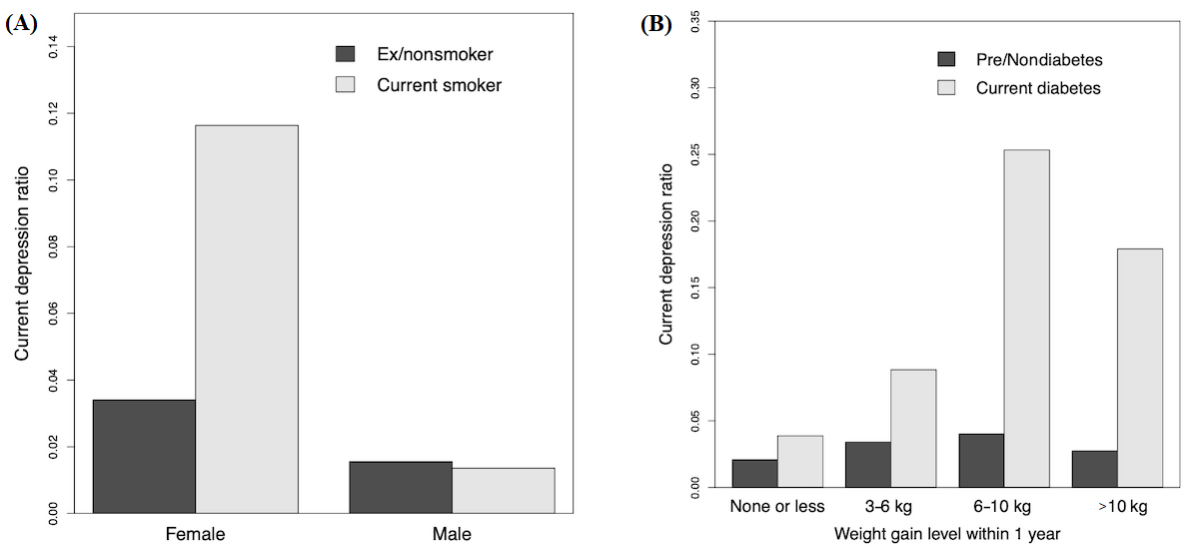
The proportion of current depression according to sex and smoking (A) and according to weight gain and diabetes (B). The interaction effects between them are significant (*P*=.001 and .01, respectively; survey-weighted logistic regression).

Another indirect factor, “current smoker,” interacted with sex and was statistically significant in females (odds ratio 2.2, 95% CI 1.3-3.7; *P*=.003) (Figure 4). Current diabetes interacted with the weight gain level that was not statistically significant for prediabetes or nondiabetes (*P*=.05) (Figure 4).

### Urine Specific Gravity and Depression

**Figure 5:**
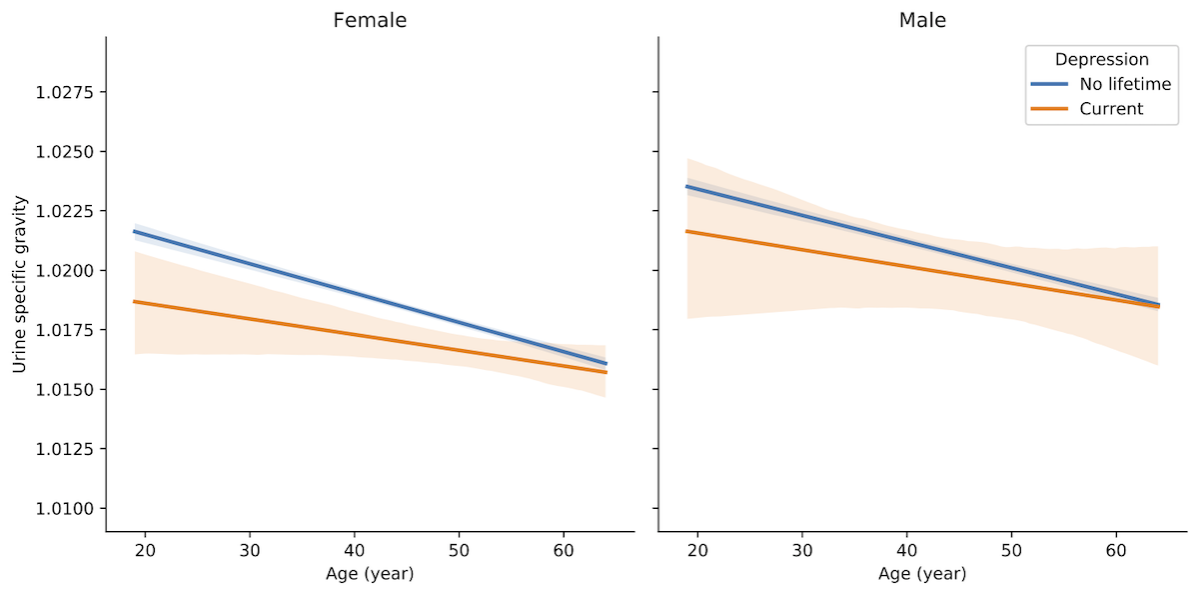
Linear regression plots between age and urine specific gravity in females and males. In the images, 95% confidence intervals for the regression estimate are drawn using translucent bands around the regression line.

**Figure 6:**
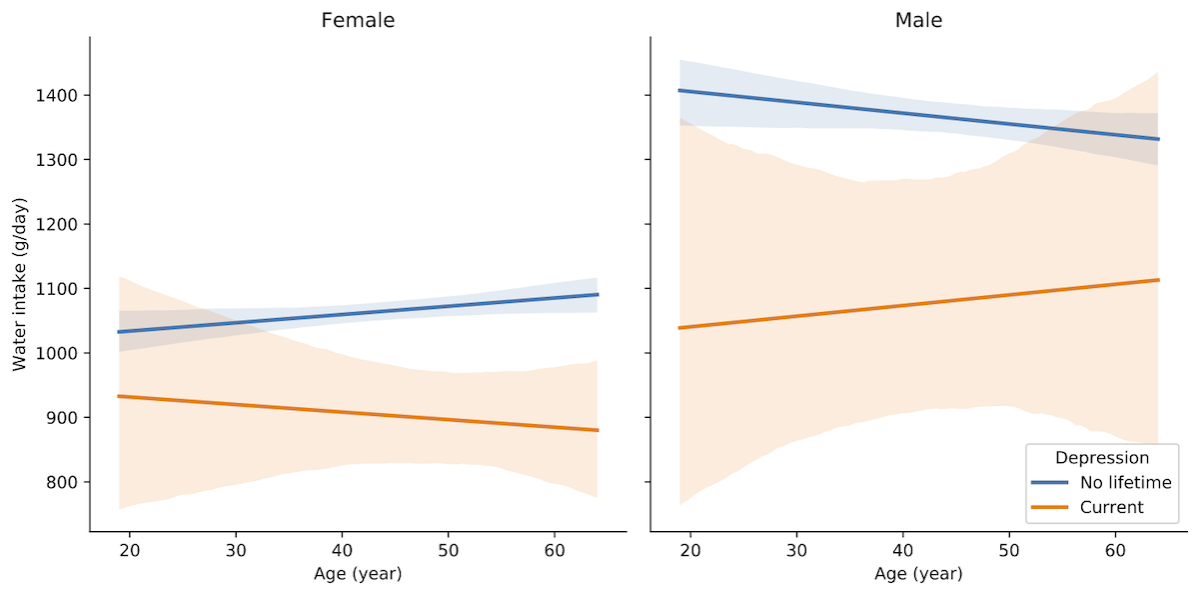
Linear regression plots between age and daily water intake in females and males. In the images, 95% confidence intervals for the regression estimate are drawn using translucent bands around the regression line.

We found an interesting relationship between urine specific gravity (USG) and depression. Because USG was associated with the “male” node on the network (Figure 3), we stratified it into males and females and plotted it according to age. USG was lower in “current depression” than in “no lifetime depression,” especially in females who were in their early 50s or younger (Figure 5). However, the daily water intake was not higher in “current depression” than in “no lifetime depression” (Figure 6).

## Discussion

### Addressing Class Imbalance and Generalization Problems

Our data set was highly imbalanced, with a 2.7% prevalence of depression. Therefore, it may be difficult for a machine learning algorithm to predict depression because this diagnosis is not sufficiently represented [28]. We used the “scale_pos_weight” parameter of XGBoost that enables class-weighted or cost-sensitive learning to make the model more sensitive to misclassification in the minority class [12]. The performance of our XGBoost model showed that the TPOT-trained class-weighted XGBoost model was working well for our imbalanced classification.

There would have been a generalization problem with this survey data because we did not train the model using the sample weight. However, the weighted AUC for the test set (0.86) was not lower than the mean unweighted AUC for the training set (0.84, SD 0.02; seven repeats of five-fold cross-validation). Therefore, our model was not overfitted, and we expect that our model can be applied to the general population.

### Reliability of Self-Reported Depression

A model may be less reliable when it is based on self-reported depression than on clinically diagnosed depression. However, for PHQ-9 scores, a clinical module, the model performance was still satisfactory when the model was retested with available cases (Figure 2). PHQ-9 is a validated screening tool for depression and is known to exhibit an acceptable diagnostic accuracy for cutoff scores between 8 and 11 [29,30]. It might be possible because we strictly defined depression by requiring both conditions (ie, current symptoms and a physician’s diagnosis). In addition, we restricted “negative depression” only to the cases when depression was never diagnosed, and respondents with a past diagnosis of depression were excluded.

### Combination of the XGBoost Model and Statistical Analysis: Feature Reduction, Indirect Factors, and Nonmodel Factors

XGBoost L1 regularization did not ultimately reduce the feature number; however, feature selection using XGBoost feature importance could decrease the feature number more at a higher reduction rate. Therefore, we suggest feature reduction using XGBoost feature importance to obtain a sparser model that contains the most principle factors of the disease.

Because of the relatively small number of predictors, a sparse model is interpretable without irrelevant features, which could be shown by the impact on the depression of each feature (Figure 2). Furthermore, we performed statistical tests on the sparse model features using multiple logistic regression at a lower risk of multicollinearity or redundancy problems. Overall, 44% of the model features were not statistically significant, and we classified them as indirect factors because they affected depression without a direct statistical association with depression.

Additionally, nonmodel factors (triglyceride, current asthma, and farm worker) exhibited statistical significance by multiple regression with depression model factors. By controlling principle depression factor effects (eg, confounding effects), nonmodel factors’ significance was reliable. Therefore, we might use a sparse disease model as a testing tool for candidate factors.

### Elements of the Depression Network: Node Size and Edge

Our network was composed of differently sized nodes (statistical strength of the association) and edges (net correlation between two factors). “Perceived stress” and “current asthma” were prioritized in depression risk factor control because of their large nodes (Figure 3). In addition to genetic factors, stressful events affect the onset of depression and cause depression through psychological stress responses, such as activating the hypothalamic-pituitary-adrenal axis [31]. Additionally, the network indicated that “perceived stress” could accompany other risk factors, such as “pain and discomfort” and “weight gain,” and we might consider them together for risk factor control (Figure 3). In contrast, current asthma was an independent node, which suggests asthma may directly link with depression (eg, asthma medications or dysregulation of specific stress-sensitive biological processes) and be an individual control target [32].

### Depression Network Centrality and Clusters: Indication of Predisposing Factors and Factor Groups

In terms of the network’s centrality indexes, “male” and “educational level” showed the highest values. Scale-free networks are characterized by growth and preferential attachment in which earlier nodes in the network increase their connectivity at a higher rate [33]. Therefore, high-centrality nodes might be preceding factors. For example, the high centrality of “educational level” might explain that a higher educational level has accumulated protective effect throughout life [34]. Therefore, the network centrality suggested that gender and the education level would be predisposed to biological and socioeconomic depression factors. Statistically, gender and the education level might be must-have covariates because of their multiple relations to other factors.

Clusters in the disease network can be potent risk intervention targets because factors in a cluster are connected and controlled together [35]. The SES cluster on our depression network is reported as the most prominent risk among psychosocial risks [35], and low SES is common in depression because of poor care, low treatment compliance, and job strain [36]. For the QoL cluster, a study reported that the usual activity problem and the pain level of EQ-5D are mainly affected by depression, and the treatment of depression improves the EQ-5D index score [37]. Depression symptoms were negatively connected to health-related QoL in the network in a previous study [38].

### Confounding and Interaction Effects of Indirect Risk Factors

If an indirect risk factor is connected to a direct risk factor, the direct factor could be the confounder. Furthermore, if the direct factor is on the causal pathway, it can be a mediator of the indirect factor action. We found potential confounders of current osteoarthritis, hypercholesterolemia, and diabetes using the network (Table 3). Osteoarthritis causes pain and physical disability, which can reduce QoL and lead to the development of depression [39]. This can be seen in our network based on the relationships among current osteoarthritis, pain, and mobility problems in the QoL cluster (Figure 3).

Obesity and metabolic syndrome may mediate the relationship between diabetes and depression [40]. This fact could be captured in our network because diabetes was associated with weight gain, higher triglycerides, and hypercholesterolemia (Figure 3). Additionally, the elevated triglyceride level was a confounder of hypercholesterolemia (Table 3), which may explain a recent meta-analysis reporting that the first episode of major depressive disorder is associated with elevated triglyceride levels, not low-density lipoprotein cholesterol or total cholesterol levels [41]. Previous studies reported that an elevated triglyceride level is associated with depression and suicidality in men [3] or women [42]. However, there was no interaction effect between “male” and triglyceride level in our study (*P*=.82).

If an indirect risk factor is connected to a direct protective factor or negatively associated with a direct risk factor, its role could be revealed by the interaction effect. “Current smoker,” which is an indirect factor strongly connected to “male,” was a significant risk factor in females, but not in males (Figures 3 and 4). Smoking cessation might be considered, especially for female depression patients [43]. Weight gain occurred in depression in current diabetes, but not clearly in prediabetes or nondiabetes (Figure 4).

### USG as a Novel Depression-Related Factor

To the best of our knowledge, USG was a novel factor of depression, and the level was low in female depressive subjects (Figure 5). USG correlates with urine osmolality and reflects the subject’s hydration status or the kidney’s concentrating ability [44]. However, our study showed that low USG in depression might not be caused by hydration because daily water intake was not low in depression (Figure 6). One study suggested that patients with depression concentrate the urine less well and excrete less solute in the urine [45].

### Limitations

This cross-sectional study involved people aged 19 to 64 years and could analyze only associations, not causalities. Therefore, the associations established in this study, such as weight gain in diabetes, elevated triglyceride, low USG, and smoking in females, require future clinical research to prove their efficacy in depression control. We used Bonferroni correction for nonmodel factors, and this conservative method might miss other possibly significant factors. Several cancers (hepatoma, gastric cancer, colon cancer, breast cancer, and lung cancer), renal failure, pulmonary tuberculosis, liver cirrhosis, and hepatitis C might not have enough positive cases to be tested. Finally, psychiatrists’ structured interviews to diagnose depression would be more valid than the self-reported approach to identify depression used in this study.

### Conclusions

We successfully created a sparse model for depression using TPOT-assisted XGBoost training and feature selection based on the feature importance of XGBoost from a large number of variables in KNHANES data. Because of the data-driven approach, we could discover a novel factor. We constructed a network of the depression-associated factors using association strength and interfactor correlations. The model factors were classified into direct and indirect according to their statistical significance, and the role of indirect factors was explained by confounding or interaction effects. The network also indicated predisposing factors by high centrality and cluster factors by a closely connected edge. Therefore, XGBoost and network analysis can be useful for discovering and understanding disease-associated factors in epidemiological studies.

## Abbreviations

AUC: area under the receiver operating characteristic curve
EBICglasso: extended Bayesian information criterium graphical lasso
EQ-5D-3L: EuroQol five-dimension three-level
KCDC: Korea Centers for Disease Control and Prevention
KNHANES: Korea National Health and Nutrition Examination Survey
LASSO: least absolute shrinkage and selection operator
PHQ-9: Patient Health Questionnaire-9
PSU: primary sampling unit
QoL: quality of life
ROC: receiver operating characteristic
SES: socioeconomic status
TPOT: tree-based pipeline optimization tool
USG: urine specific gravity
XGBoost: extreme gradient boosting
